# Blood cytology in children with down syndrome

**DOI:** 10.1186/s12887-022-03450-8

**Published:** 2022-07-02

**Authors:** Silvestre García de la Puente, Karla Adney Flores-Arizmendi, Yessica Yuliana Guerrero-Tapia, Tania Tonantzin Vargas-Robledo, Norma Candelaria López-Santiago

**Affiliations:** 1 Department of Research Methodology, National Institute of Pediatrics, Insurgentes Sur 3700-C, Colonia Insurgentes Cuicuilco, Alcaldía Coyoacán, 04530 México City, CDMX Mexico; 2 Down Syndrome Clinic, National Institute of Pediatrics, Mexico City, Mexico; 3 Hematology Service, National Institute of Pediatrics, Mexico City, Mexico

**Keywords:** Down syndrome, Blood cells, Children, Not-malignant hematological alterations

## Abstract

**Introduction:**

Down syndrome is associated with various congenital anomalies and metabolic alterations such as hematological alterations. Values for the major hematological indicators vary with age and sex, but these values have not been described for Mexican children with Down syndrome.

**Objective:**

To describe the complete blood count (CBC) values of pediatric patients with Down syndrome in México and report the most common non-malignant hematological alterations.

**Materials and methods:**

The analysis includes data from 450 patients with Down syndrome, 55.5% ware males, aged 0-18 years who were patients at the Mexican National Institute of Pediatrics and whose clinical charts included CBC panel results for the period January 2008 through March 2018.

**Results:**

A total of 3438 CBC panels were analyzed with descriptive statistics to find the values and statistical dispersion of the major indicators, with percentiles, and reported separately by sex and age group. The most common non-malignant hematological alterations found were macrocytic anemia, leukopenia, lymphopenia, and thrombocytosis. There were differences in values in all three series.

**Conclusions:**

The CBC panels and hematological alterations are summarized for patients with Down syndrome.

**Supplementary Information:**

The online version contains supplementary material available at 10.1186/s12887-022-03450-8.

## Introduction

Down syndrome is the most common human genetic disorder [1]. In Mexico, the Secretary of Health estimates its prevalence at 1 in 650 live newborns [2]. This chromosomal anomaly causes intellectual disability, specific phenotypic characteristics, and a combination of systemic conditions [1, 2]. Among the hematological alterations seen are leukemia, transient abnormal myelopoiesis, neutrophilia, and thrombocytopenia with giant platelets [3–7]. Macrocytosis, thrombocytosis, and leukopenia have been reported in older children [8, 9].

Values for the major hematological indicators vary with age and sex [10, 11], but these values have not been described for Mexican children with Down syndrome. The objective of this study is to do so, as well as to describe the non-malignant hematological alterations found in this population.

This study was approved by the Research and Investigation Committees of the National Institute of Pediatrics (Approval No. 2018/025).

## Materials and methods

This was an observational, descriptive, cross-sectional, and retrospective study, carried out in the Down Syndrome Clinic of the National Institute of Pediatrics. Data were taken from the medical charts of patients aged 0-18 years, both sexes, which included complete blood count (CBC) panels as part of their ongoing care, for the period January 2008 to March 2018. The following values were taken from the clinical laboratory database used by this institution (Medsys): hemoglobin, hematocrit, erythrocytes, red cell distribution width (RDW), mean corpuscular volume (MCV), mean corpuscular hemoglobin (MCH), mean corpuscular hemoglobin concentration (MCHC), leukocytes, neutrophils, lymphocytes, basophils, monocytes, eosinophils, platelets, and mean platelet volume (MPV). CBC analyses were analyzed according to the manufacturer’s specification, with a Beckman Coulter DxH 900 hematology analyzer.

Patients with suspected or confirmed diagnoses of complex cardiopathy or oncological or immunological illness were excluded, as were panels for included patients taken during an infectious process, either during hospitalization or an outpatient visit. Patient records were divided by sex and into five groups by age at the time blood was taken (< 2 years, 2-5 years, 6-11 years, 12-14 years, and 15-18 years), and descriptive statistics were obtained. The following benign hematological alterations were also noted, based on values that were out of the normal range for the patient’s age and sex in the Mexican pediatric population: anemia, macrocytosis, microcytosis, leukocytosis, leukopenia, neutrophilia, neutropenia, lymphocytosis, lymphopenia, eosinophilia, eosinopenia, thrombocytosis, and thrombocytopenia [12].

## Results

This study reviewed 496 patient charts from the Down Syndrome Clinic of the National Institute of Pediatrics, 46 subjects were excluded because of incomplete information. Of the remaining 450, 200 were for girls and 250 were for boys. Cytogenic confirmation was performed for 87%, with findings of regular trisomy in 80%, mosaic trisomy in 4%, and translocation trisomy in 3%. A total of 3438 CBC panels were grouped by sex and age at the time the sample was taken. Table 1 shows the number of samples by sex and age; Table 2 summarizes means, standard deviations, and 95% confidence intervals for each indicator by sex and age group. A factorial ANOVA was performed to analyze differences in indicator values by age and sex, and differences were generally found. Table 3 shows the statistical significance of the analysis, which can more easily be visualized graphically in Fig. S1 in Online Resource 1. Hemoglobin increases with age and is greatest in boys. Neutrophils increase and lymphocytes decrease with age. Platelets decrease with age and are slightly higher in boys.

**Table 1:** Sample distribution by sex and age

Sex and age	***n***	%
**Male**	**1953**	**56.8**
< 2 years	473	13.8
2-5 years	760	22.1
6-11 years	562	16.3
12-14 years	115	3.3
15-18 years	43	1.3
**Female**	**1485**	**43.2**
< 2 years	291	8.4
2-5 years	519	15.1
6-11 years	462	13.4
12-14 years	116	3.4
15-18 years	97	2.8

**Table 2:** Complete Blood Count (CBC) reference values by sex and age

Indicator	*n*	Sex	Age	Mean	Standard Deviation	95% CI
Hemoglobin (g/dL)	473	Male	< 2	13.1	1.4	12.97, 13.23
Hemoglobin (g/dL)	760	Male	2-5	14.12	1.18	14.04, 14.2
Hemoglobin (g/dL)	562	Male	6-11	14.66	1.08	14.57, 14.74
Hemoglobin (g/dL)	115	Male	12-14	15.47	1.24	15.24, 15.7
Hemoglobin (g/dL)	43	Male	15-18	15.86	0.73	15.63, 16.08
Hemoglobin (g/dL)	291	Female	< 2	13.05	1.58	12.87, 13.23
Hemoglobin (g/dL)	519	Female	2-5	14.05	1.15	13.95, 14.15
Hemoglobin (g/dL)	462	Female	6-11	14.75	1.1	14.65, 14.85
Hemoglobin (g/dL)	116	Female	12-14	15.18	0.88	15.01, 15.34
Hemoglobin (g/dL)	97	Female	15-18	15.27	0.87	15.1, 15.45
Hematocrit (%)	473	Male	< 2	39.08	4.18	38.7, 39.45
Hematocrit (%)	760	Male	2-5	41.62	3.43	41.38, 41.86
Hematocrit (%)	562	Male	6-11	43.11	3.01	42.86, 43.36
Hematocrit (%)	115	Male	12-14	45.55	3.43	44.92, 46.18
Hematocrit (%)	43	Male	15-18	46.81	2.12	46.16, 47.46
Hematocrit (%)	291	Female	< 2	39.12	4,89	38.56, 39.69
Hematocrit (%)	519	Female	2-5	41.53	3.36	41.24, 41.82
Hematocrit (%)	462	Female	6-11	43.34	3.16	43.06, 43.63
Hematocrit (%)	116	Female	12-14	44.68	2.78	44.17, 45.19
Hematocrit (%)	97	Female	15-18	45.22	2.54	44.71, 45.73
Erythrocytes (10^˄^6/μL)	473	Male	< 2	4.3	0.53	4.26, 4.35
Erythrocytes (10^˄^6/μL)	760	Male	2-5	4.55	0.43	4.52, 4.58
Erythrocytes (10^˄^6/μL)	562	Male	6-11	4.64	0.38	4.61,4.67
Erythrocytes (10^˄^6/μL)	115	Male	12-14	4.88	0.36	4.81, 4.94
Erythrocytes (10^˄^6/μL)	43	Male	15-18	4.98	0.27	4.9, 5.06
Erythrocytes (10^˄^6/μL)	291	Female	< 2	4.32	0.61	4.25, 4.39
Erythrocytes (10^˄^6/μL)	519	Female	2-5	4.53	0.39	4.5, 4.57
Erythrocytes (10^˄^6/μL)	462	Female	6-11	4.62	0.37	4.59, 4.66
Erythrocytes (10^˄^6/μL)	116	Female	12-14	4.68	0.36	4.62, 4.74
Erythrocytes (10^˄^6/μL)	97	Female	15-18	4.7	0.29	4.64, 4.75
RDW (%)	473	Male	< 2	15.5	2.04	15.32, 15.69
RDW (%)	760	Male	2-5	14.72	1.45	14.62. 14.82
RDW (%)	562	Male	6-11	14.42	1.26	14.31, 14.52
RDW (%)	115	Male	12-14	14.82	1.83	14.48, 15.15
RDW (%)	43	Male	15-18	14.66	1.59	14.17. 15.15
RDW (%)	291	Female	< 2	16.1	2.94	15.76, 16.44
RDW (%)	519	Female	2-5	14.68	1.61	14.54, 14.82
RDW (%)	462	Female	6-11	14.06	0.95	13.97, 14.15
RDW (%)	116	Female	12-14	14.05	0.87	13.89, 14.21
RDW (%)	97	Female	15-18	13.93	0.77	13.77, 14.08
MCV (fL)	473	Male	< 2	91.17	6.09	90.62, 91.72
MCV (fL)	760	Male	2-5	91.74	4.86	91.4, 92.09
MCV (fL)	562	Male	6-11	93.05	4.32	92.69, 93.4
MCV (fL)	115	Male	12-14	93.48	5.16	92.53, 94.43
MCV (fL)	43	Male	15-18	94.16	4.91	92.65. 95.67
MCV (fL)	291	Female	< 2	91.02	6.57	90.26, 91.78
MCV (fL)	519	Female	2-5	91.76	4.58	91.37, 92.16
MCV (fL)	462	Female	6-11	93.87	3.56	93.55, 94.2
MCV (fL)	116	Female	12-14	95.55	3.57	94.89, 96.21
MCV (fL)	97	Female	15-18	96.36	3.24	95.71, 97.02
MCH (pg)	473	Male	< 2	30.57	2.27	30.37. 30.78
MCH (pg)	760	Male	2-5	31.13	1.9	31.0, 31. 27
MCH (pg)	562	Male	6-11	31.64	1.71	31.5, 31.78
MCH (pg)	115	Male	12-14	31.75	2.11	31.36, 32.14
MCH (pg)	43	Male	15-18	31.91	1.92	31.32, 32.5
MCH (pg)	291	Female	< 2	30.39	2.46	30.1, 30.67
MCH (pg)	519	Female	2-5	31.05	1.84	30.89, 31.21
MCH (pg)	462	Female	6-11	31.95	1.48	31.82, 32.09
MCH (pg)	116	Female	12-14	32.48	1.58	32.19, 32.77
MCH (pg)	97	Female	15-18	32.57	1.46	32.27, 32.88
MCHC (g/dL)	473	Male	< 2	33.53	0.93	33.44, 33.61
MCHC (g/dL)	760	Male	2-5	33.93	0.84	33.87, 33.99
MCHC (g/dL)	562	Male	6-11	34	0.84	33.93, 34.06
MCHC (g/dL)	115	Male	12-14	33.95	0.85	33.8, 34.11
MCHC (g/dL)	43	Male	15-18	33.88	0.69	33.67, 34.09
MCHC (g/dL)	291	Female	< 2	33.38	1.16	33.25, 33.51
MCHC (g/dL)	519	Female	2-5	33.83	0.97	33.75, 33.92
MCHC (g/dL)	462	Female	6-11	34.03	0.73	33.97, 34.1
MCHC (g/dL)	116	Female	12-14	33.99	0.8	33.84, 34.13
MCHC (g/dL)	97	Female	15-18	33.79	0.73	33.64, 33.94
Leukocytes (10˄3/μL)	473	Male	< 2	6.86	1.72	6.7, 7.01
Leukocytes (10˄3/μL)	760	Male	2-5	6.32	1.74	6.2, 6.44
Leukocytes (10˄3/μL)	562	Male	6-11	5.79	1.67	5.65, 5.93
Leukocytes (10˄3/μL)	115	Male	12-14	5.17	1.74	4.85, 5.49
Leukocytes (10˄3/μL)	43	Male	15-18	6.08	1.79	5.53, 6.63
Leukocytes (10˄3/μL)	291	Female	< 2	6.87	2.03	6.64, 7.11
Leukocytes (10˄3/μL)	519	Female	2-5	6.02	1.71	5.88, 6.17
Leukocytes (10˄3/μL)	462	Female	6-11	5.46	1.65	5.31, 5.61
Leukocytes (10˄3/μL)	116	Female	12-14	6.09	1.82	5.76, 6.43
Leukocytes (10˄3/μL)	97	Female	15-18	6.23	1.64	5.9, 6.56
Neutrophils (%)	473	Male	< 2	42.69	15.17	41.32, 44.06
Neutrophils (%)	760	Male	2-5	50.43	14.36	49.41, 51.46
Neutrophils (%)	562	Male	6-11	52.,53	12.69	51.48, 53.58
Neutrophils (%)	115	Male	12-14	53.04	12.43	50.74, 55.34
Neutrophils (%)	43	Male	15-18	55.19	9.15	52.37, 58.0
Neutrophils (%)	291	Female	< 2	42.34	16.99	40.38, 44.3
Neutrophils (%)	519	Female	2-5	49.94	14.47	48.7, 51.19
Neutrophils (%)	462	Female	6-11	54.19	11.98	53.1, 55.29
Neutrophils (%)	116	Female	12-14	57.77	11.95	55.57, 59.97
Neutrophils (%)	97	Female	15-18	57.76	11.28	55.48, 60.03
Lymphocytes (%)	473	Male	< 2	46.02	13.9	44.76, 47.27
Lymphocytes (%)	760	Male	2-5	38.92	13.21	37.98, 39.86
Lymphocytes (%)	562	Male	6-11	37.0	12.41	35.97, 38.03
Lymphocytes (%)	115	Male	12-14	36.27	11.89	34.08, 38.47
Lymphocytes (%)	43	Male	15-18	34.55	8.8	31.84, 37.26
Lymphocytes (%)	291	Female	< 2	46.59	15.53	44.79, 48.38
Lymphocytes (%)	519	Female	2-5	40.13	13.35	38.98, 41.28
Lymphocytes (%)	462	Female	6-11	35.8	11.27	34.78, 36.84
Lymphocytes (%)	116	Female	12-14	32.38	11.55	30.26, 34.51
Lymphocytes (%)	97	Female	15-18	32.44	10.87	30.25, 34.63
Monocytes (%)	473	Male	< 2	8.52	3.8	8.17, 8.86
Monocytes (%)	760	Male	2-5	8.22	3.15	8.0, 8.44
Monocytes (%)	562	Male	6-11	8.09	2.47	7.89, 8.3
Monocytes (%)	115	Male	12-14	8.3	2.39	7.86, 8.74
Monocytes (%)	43	Male	15-18	8.5	2.19	7.83, 9.17
Monocytes (%)	291	Female	< 2	8.62	4.29	8.13, 9.12
Monocytes (%)	519	Female	2-5	7.7	2.86	7.45, 7.95
Monocytes (%)	462	Female	6-11	7.75	2.39	7.53, 7.97
Monocytes (%)	116	Female	12-14	7.66	2.0	7.3, 8.03
Monocytes (%)	97	Female	15-18	7.54	1.76	7.19, 7.9
Eosinophils (%)	473	Male	< 2	1.9	1.91	1.73, 2.07
Eosinophils (%)	760	Male	2-5	1.69	1.83	1.56, 1.82
Eosinophils (%)	562	Male	6-11	1.55	1.11	1.45, 1.64
Eosinophils (%)	115	Male	12-14	1.54	1.22	1.32, 1.77
Eosinophils (%)	43	Male	15-18	1.04	0.75	0.81, 1.27
Eosinophils (%)	291	Female	< 2	1.51	1.64	1.32, 1.7
Eosinophils (%)	519	Female	2-5	1.48	1.57	1.34, 1.61
Eosinophils (%)	462	Female	6-11	1.47	1.61	1.32, 1.61
Eosinophils (%)	116	Female	12-14	1.44	0.97	1.26, 1.62
Eosinophils (%)	97	Female	15-18	1.54	1.11	1.32, 1.76
Basophils (%)	473	Male	< 2	0.73	0.72	0.67, 0.8
Basophils (%)	760	Male	2-5	0.78	0.59	0.74, 0.82
Basophils (%)	562	Male	6-11	0.81	0.46	0.77, 0.85
Basophils (%)	115	Male	12-14	0.85	0.41	0.77, 0.92
Basophils (%)	43	Male	15-18	0.72	0.3	0.63, 0.81
Basophils (%)	291	Female	< 2	0.72	0.82	0.63, 0.82
Basophils (%)	519	Female	2-5	0.71	0.68	0.65, 0.77
Basophils (%)	462	Female	6-11	0.72	0.43	0.68, 0.76
Basophils (%)	116	Female	12-14	0.69	0.42	0.61, 0.76
Basophils (%)	97	Female	15-18	0.72	0.38	0.65, 0.8
Neutrophils (10˄3/μL)	473	Male	< 2	2.99	1.49	2.86, 3.13
Neutrophils (10˄3/μL)	760	Male	2-5	3.26	1.51	3.15, 3.37
Neutrophils (10˄3/μL)	562	Male	6-11	3.09	1.35	2.98, 3.2
Neutrophils (10˄3/μL)	115	Male	12-14	2.84	1.4	2.58, 3.1
Neutrophils (10˄3/μL)	43	Male	15-18	3.45	1.41	3.01, 3.88
Neutrophils (10˄3/μL)	291	Female	< 2	3.0	1.65	2.81, 3.19
Neutrophils (10˄3/μL)	519	Female	2-5	3.11	1.56	2.97, 3.24
Neutrophils (10˄3/μL)	462	Female	6-11	3.05	1.44	2.92, 3.18
Neutrophils (10˄3/μL)	116	Female	12-14	3.57	1.5	3.3, 3.85
Neutrophils (10˄3/μL)	97	Female	15-18	3.68	1.41	3.4, 3.96
Lymphocytes (10˄3/μL)	473	Male	< 2	3.1	1.14	3.0, 3.2
Lymphocytes (10˄3/μL)	760	Male	2-5	2.41	1.0	2.34, 2.48
Lymphocytes (10˄3/μL)	562	Male	6-11	2.11	0.93	2.03, 2.19
Lymphocytes (10˄3/μL)	115	Male	12-14	1.8	0.72	1.66, 1.93
Lymphocytes (10˄3/μL)	43	Male	15-18	2.02	0.56	1.85, 2.19
Lymphocytes (10˄3/μL)	291	Female	< 2	3.13	1.26	2.99, 3.28
Lymphocytes (10˄3/μL)	519	Female	2-5	2.34	0.9	2.26, 2.41
Lymphocytes (10˄3/μL)	462	Female	6-11	1.88	0.68	1.82, 1.95
Lymphocytes (10˄3/μL)	116	Female	12-14	1.94	0.93	1.76, 2.1
Lymphocytes (10˄3/μL)	97	Female	15-18	1.95	0.66	1.82, 2.08
Monocytes (10˄3/μL)	473	Male	< 2	0.57	0.29	0.55, 0.6
Monocytes (10˄3/μL)	760	Male	2-5	0.51	0.22	0.49, 0.52
Monocytes (10˄3/μL)	562	Male	6-11	0.46	0.18	0.44, 0.48
Monocytes (10˄3/μL)	115	Male	12-14	0.42	0.16	0.39, 0.45
Monocytes (10˄3/μL)	43	Male	15-18	0.51	0.2	0.45, 0.57
Monocytes (10˄3/μL)	291	Female	< 2	0.58	0.31	0.54, 0.61
Monocytes (10˄3/μL)	519	Female	2-5	0.45	0.2	0.43, 0.47
Monocytes (10˄3/μL)	462	Female	6-11	0.41	0.15	0.4, 0.42
Monocytes (10˄3/μL)	116	Female	12-14	0.46	0.17	0.43, 0.49
Monocytes (10˄3/μL)	97	Female	15-18	0.46	0.16	0.43, 0.49
Eosinophils (10˄3/μL)	473	Male	< 2	0.13	0.14	0.12, 0.14
Eosinophils (10˄3/μL)	760	Male	2-5	0.1	0.12	0.09, 0.11
Eosinophils (10˄3/μL)	562	Male	6-11	0.09	0.07	0.08, 0.09
Eosinophils (10˄3/μL)	115	Male	12-14	0.08	0.07	0.07, 0.09
Eosinophils (10˄3/μL)	43	Male	15-18	0.06	0.05	0.05, 0.08
Eosinophils (10˄3/μL)	291	Female	< 2	0.1	0.12	0.09. 0.11
Eosinophils (10˄3/μL)	519	Female	2-5	0.08	0.09	0.07, 0.09
Eosinophils (10˄3/μL)	462	Female	6-11	0.08	0.09	0.07, 0.08
Eosinophils (10˄3/μL)	116	Female	12-14	0.08	0.05	0.07, 0.09
Eosinophils (10˄3/μL)	97	Female	15-18	0.09	0.07	0.08, 0.11
Basophils (10˄3/μL)	473	Male	< 2	0.05	0.05	0.04, 0.05
Basophils (10˄3/μL)	760	Male	2-5	0.05	0.04	0.04, 0.05
Basophils (10˄3/μL)	562	Male	6-11	.05	0.03	0.04, 0.05
Basophils (10˄3/μL)	115	Male	12-14	0.04	0.03	0.04, 0.05
Basophils (10˄3/μL)	43	Male	15-18	0.04	0.02	0.04, 0.05
Basophils (10˄3/μL)	291	Female	< 2	0.05	0.06	0.04, 0.06
Basophils (10˄3/μL)	519	Female	2-5	0.04	0.05	0.04, 0.05
Basophils (10˄3/μL)	462	Female	6-11	0.04	0.02	0.04, 0.04
Basophils (10˄3/μL)	116	Female	12-14	0.04	0.03	0.03, 0.04
Basophils (10˄3/μL)	97	Female	15-18	0.04	0.03	0.04, 0.05
Platelets (10˄3/μL)	473	Male	< 2	324.43	81.37	317.1, 331.8
Platelets (10˄3/μL)	760	Male	2-5	306.62	70.98	301.6, 311.7
Platelets (10˄3/μL)	562	Male	6-11	293.89	65.54	288.5, 299.3
Platelets (10˄3/μL)	115	Male	12-14	272.24	56.53	261.8, 282.7
Platelets (10˄3/μL)	43	Male	15-18	266.56	59.88	248.1, 285
Platelets (10˄3/μL)	291	Female	< 2	310.44	86.61	300.5, 320.4
Platelets (10˄3/μL)	519	Female	2-5	300.01	76.16	293.4, 306.6
Platelets (10˄3/μL)	462	Female	6-11	280.16	66.36	274.1, 286.2
Platelets (10˄3/μL)	116	Female	12-14	260.3	65.9	248.2, 272.4
Platelets (10˄3/μL)	97	Female	15-18	255.16	64.45	242.2, 268.2
MPV (fL)	473	Male	< 2	7.79	0.83	7.71, 7.86
MPV (fL)	760	Male	2-5	7.44	0.74	7.39, 7.5
MPV (fL)	562	Male	6-11	7.53	0.64	7.48, 7.58
MPV (fL)	115	Male	12-14	7.69	0.65	7.57, 7.81
MPV (fL)	43	Male	15-18	7.78	0.8	7.54, 8.03
MPV (fL)	291	Female	< 2	7.79	0.91	7.68, 7.89
MPV (fL)	519	Female	2-5	7.39	0.69	7.33, 7.45
MPV (fL)	462	Female	6-11	7.39	0.6	7.33, 7.44
MPV (fL)	116	Female	12-14	7.58	0.57	7.48, 7.68
MPV (fL)	97	Female	15-18	7.63	0.58	7.51, 7.74

*Abbreviations: RDW* Red cell distribution width, *MCV* Mean corpuscular volume, *MCH* Mean corpuscular hemoglobin, *MCHC* Mean corpuscular hemoglobin concentration, *MPV* Mean platelet volume

**Table 3:** Differences by sex and age in CBC values

Indicator	Sex *p*	Age *p*	Sex*Age *p*
Hemoglobin (g/dL)	0.003	<0.001	0.016
Hematocrit (%)	0.011	<0.001	0.032
Erythrocytes (10^˄^6/μL)	<0.001	<0.001	<0.001
RDW (%)	0.002	<0.001	<0.001
MCV (fL)	<0.001	<0.001	0.002
MCH (pg)	0.003	<0.001	0.001
MCHC (g/dL)	0.249	<0.001	0.197
Leukocytes (10˄3/μL)	0.301	<0.001	<0.001
Neutrophils (%)	0.021	<0.001	0.043
Lymphocytes (%)	0.098	<0.001	0.026
Monocytes (%)	0.002	<0.001	0.149
Eosinophils (%)	0.471	0.025	0.043
Basophils (%)	0.032	0.686	0.438
Neutrophils (10˄3/μL)	0.038	0.001	0.001
Lymphocytes (10˄3/μL)	0.432	<0.001	0.024
Monocytes (10˄3/μL)	0.187	<0.001	0.002
Eosinophils (10˄3/μL)	0.313	<0.001	0.036
Basophils (10˄3/μL)	0.147	<0.001	0.259
Platelets (10˄3/μL)	0.002	<0.001	0.77
MPV (fL)	0.012	<0.001	0.318

Analysis: Factorial ANOVA

Table 4 shows the hematological alterations found, with reference to the reported ranges by age group for the Mexican population [12]. These included 129 samples (3.8%) with a diagnosis of anemia, of which 11 were microcytic, 60 macrocytic, and the rest normocytic. Of particular note is the high frequency of elevated mean corpuscular volume, which was seen in 2076 samples (60.4%), most without anemia or polycythemia. The white blood cell series showed leukopenia in 368 samples (10.7%), lymphopenia in 807 (23.5%), eosinopenia in 341 (9.9%), basophilia in 274 (8%), neutrophilia in 226 (6.6%), and neutropenia in 118 (3.4%). Thrombocytosis was found in 350 (10.2%) and thrombocytopenia in 71 (2.1%).

**Table 4:** Hematological alterations

Alteration	***n***	%
**Anemia**	**129**	**3.8**
**Microcytosis**	**29**	**0.8**
**Macrocytosis**	**2076**	**60.4**
**Hyperchromia**	**427**	**12.4**
**Hypochromia**	**79**	**2.3**
**Leukocytosis**	**10**	**0.3**
**Leukopenia**	**368**	**10.7**
**Lymphopenia**	**807**	**23.5**
**Eosinopenia**	**341**	**9.9**
**Basophilia**	**274**	**8**
**Neutrophilia**	**226**	**6.6**
**Neutropenia**	**118**	**3.4**
**Thrombocytopenia**	**71**	**2.1**
**Thrombocytosis**	**350**	**10.2**

Tables S1 and S2 in Online Resource 2 show the percentiles by age and sex for the different indicators.

## Discussion

CBC panel is one of the most common used diagnostic tests in medical practice. When we review it, we should consider that pediatric reference values are different than those for adults, and that they will vary with such factors as age, sex, and height. We should also consider that the reference values include 95% of the “normal” population: that is, the populational mean ± 1.96 standard deviations. Values outside this range do not always imply pathology [13]. Studies have been conducted to determine the CBC reference ranges for the Mexican pediatric population, but there has been no study to determine these ranges for the population with Down syndrome.

Among the systemic alterations associated with Down syndrome are hematological problems. Newborns with Down syndrome have a greater risk for transient neonatal leukemia, known as transient abnormal myelopoiesis (TAM), characterized by circulating blasts with morphological and phenotypic characteristics of leukemic cells. TAM is estimated to occur in approximately 10% of newborns with Down syndrome. Although it is transient, a small number develop serious or even fatal complications such as hepatic fibrosis or edema, and 20% of those who recover from TAM develop acute megakaryoblastic leukemia [5].

Newborns with Down syndrome may have normal blood counts, although they can show subtle anomalies and dysplastic characteristics of the white corpuscles, platelets, and red corpuscles. These characteristics are often described as incidental findings. Although they are probably not clinically important, the early recognition of non-malignant alterations in these patients is important. To date there are no reference values for different age groups in this population; those reported for the general population have been used in an arbitrary way.

Other hematological alterations that have been found in newborns with Down syndrome include neutrophilia, thrombocytopenia, and polycythemia, with incidences of 80, 66, and 34%, respectively, reported by Henry et al. [7]. These anomalies generally have a benign clinical course and resolve spontaneously at three weeks of age [3]. The neutrophilia is slight, rarely over 30.000/μL, and is not associated with an infection. The thrombocytopenia is also slight, the majority with platelet counts < 150,000/μL, and is not associated with hemorrhage. Kivivuori et al. [9]. carried out a prospective follow-up study of the platelet counts of twenty-five newborns with Down syndrome during their first year of life and found that thrombocytopenia is generally brief, resolves itself in the first few weeks, and is later replaced by thrombocytosis. Polycythemia is usually slight, and only some presented cyanosis, which is corrected with partial exchange transfusion. It is usually independent of the heart defects and hypoxia that are often associated with this condition.

Our study included only twenty-six newborns; none of them presented polycythemia, only one with neutrophilia (3.8%), and three with thrombocytopenia (11.5%), a contrast with the findings of Henry et al. [7]. However, the small size of our sample in this age group must be considered.

At later ages, anemia, macrocytosis, and leukopenia were common in our sample, at levels like those previously reported by Roizen and Kivivuori [8, 9]. High levels of mean corpuscular volume have been reported in patients with Down syndrome at all ages. David et al. [14]. found no underlying cause for macrocytosis and postulated an altered folate remethylation path as an effect of greater cystathionine-β-synthase activity. Our findings coincide with these studies. Evaluation of a patient with anemia should consider its possible underestimation due to macrocytosis, and consider the use of other tools, such as analysis of folates, vitamin B12, and iron for greater diagnostic certainty.

Finally, we observed some hematological alterations not previously reported for patients with Down syndrome: lymphopenia, eosinopenia, basophilia, neutrophilia, and neutropenia. Although these have no clinical significance, they should be monitored.

## Conclusion

This study provides us with a broader overview of the parameters that should be considered in the analysis of blood counts for the pediatric population with Down syndrome. We believe it can be taken as a reference for evaluation and routine follow-up, as well as a reminder that these patients may have non-malignant hematological alterations that require monitoring.

## Supplementary Information

**Additional file 1.**

**Additional file 2.**

## Data Availability

All data generated or analyzed during this study are included in this published article [and its supplementary information files].
