# Supplementary material for: Blood cytology in children with down syndrome
Source: BMC Pediatr. 2022 Jul 2;22:387. doi: 10.1186/s12887-022-03450-8 (PMC9250261; doi:10.1186/s12887-022-03450-8)
Supplement: Supplementary file 1 — Additional file 1. [file 12887_2022_3450_MOESM1_ESM.docx]

**Table S1. Male Percentiles**

| **Indicator** | **Age (years)** | **Percentile** | | | | | | |
| --- | --- | --- | --- | --- | --- | --- | --- | --- |
| **Hemoglobin** |  | **5** | **10** | **25** | **50** | **75** | **90** | **95** |
| g/dL | **˂2** | 10.60 | 11.30 | 12.20 | 13.10 | 14.00 | 14.80 | 15.40 |
|  | **2-5** | 12.10 | 12.70 | 13.40 | 14.20 | 14.90 | 15.50 | 15.90 |
|  | **6-11** | 12.80 | 13.30 | 14.10 | 14.80 | 15.40 | 15.80 | 16.10 |
|  | **12-14** | 13.02 | 14.10 | 14.90 | 15.70 | 16.40 | 16.74 | 16.90 |
|  | **15-18** | 14.82 | 15.04 | 15.40 | 15.90 | 16.30 | 16.90 | 17.00 |
| **Hematocrit %** | **˂2** | 31.57 | 33.64 | 36.80 | 39.00 | 41.60 | 44.24 | 46.20 |
|  | **2-5** | 35.82 | 37.31 | 39.6 | 41.90 | 43.90 | 45.7 | 46.8 |
|  | **6-11** | 38.12 | 39.40 | 41.30 | 43.40 | 45.20 | 46.60 | 47.30 |
|  | **12-14** | 39.06 | 41.88 | 43.70 | 46.00 | 48.00 | 49.6 | 50.12 |
|  | **15-18** | 43.74 | 44.04 | 45.10 | 47.20 | 48.40 | 49.70 | 50.12 |
| ***Erythrocytes*** | **˂2** | 3.35 | 3.55 | 3.99 | 4.32 | 4.65 | 4.99 | 5.15 |
| 10^˄^6/μL | **2-5** | 3.86 | 4.02 | 4.28 | 4.57 | 4.84 | 5.09 | 5.23 |
|  | **6-11** | 3.99 | 4.18 | 4.39 | 4.65 | 4.90 | 5.12 | 5.26 |
|  | **12-14** | 4.30 | 4.47 | 4.66 | 4.84 | 5.19 | 5.35 | 5.48 |
|  | **15-18** | 4.61 | 4.64 | 4.74 | 4.93 | 5.18 | 5.37 | 5.49 |
| **RDW** | **˂2** | 13.20 | 13.50 | 14.10 | 15.10 | 16.40 | 18.40 | 19.63 |
| **%** | **2-5** | 13.20 | 13.40 | 13.80 | 14.40 | 15.20 | 16.40 | 17.40 |
|  | **6-11** | 13.20 | 13.40 | 13.70 | 14.20 | 14.70 | 15.50 | 16.58 |
|  | **12-14** | 13.30 | 13.46 | 13.80 | 14.30 | 15.10 | 16.54 | 19.10 |
|  | **15-18** | 13.26 | 13.50 | 13.90 | 14.30 | 14.90 | 15.36 | 18.14 |
| **MCV** | **˂2** | 82.11 | 84.80 | 87.90 | 91.30 | 94.20 | 98.60 | 100.93 |
| **fL** | **2-5** | 84.10 | 86.10 | 88.90 | 91.80 | 95.00 | 97.69 | 99.70 |
|  | **6-11** | 86.53 | 88.00 | 90.10 | 92.80 | 95.70 | 98.97 | 101.0 |
|  | **12-14** | 83.34 | 87.12 | 90.30 | 93.70 | 96.50 | 100.74 | 102.74 |
|  | **15-18** | 82.54 | 85.44 | 91.80 | 94.80 | 97.20 | 99.12 | 100.2 |
| **MCH**  **pg** | **˂2** | 26.97 | 28.00 | 29.35 | 30.60 | 31.80 | 33.40 | 34.23 |
|  | **2-5** | 28.30 | 28.90 | 30.00 | 31.10 | 32.40 | 33.40 | 34.20 |
|  | **6-11** | 28.91 | 29.60 | 30.50 | 31.60 | 32.60 | 33.80 | 34.78 |
|  | **12-14** | 26.98 | 28.78 | 31.10 | 31.00 | 33.00 | 34.30 | 35.18 |
|  | **15-18** | 27.60 | 28.94 | 31.00 | 32.20 | 33.00 | 34.26 | 34.40 |
| **MCHC**  **g/dL** | **˂2** | 31.9 | 32.40 | 32.90 | 33.50 | 34.1 | 34.6 | 35.00 |
|  | **2-5** | 32.60 | 32.90 | 33.40 | 33.90 | 34.50 | 34.90 | 35.20 |
|  | **6-11** | 32.7 | 33.00 | 33.50 | 34.00 | 34.40 | 35.00 | 35.30 |
|  | **12-14** | 32.30 | 32.76 | 33.50 | 34.00 | 34.50 | 35.00 | 35.22 |
|  | **15-18** | 32.22 | 33.14 | 33.40 | 33.90 | 34.40 | 34.76 | 34.88 |
| **Leukocytes** 10˄3/μL | **˂2** | 3.97 | 4.50 | 5.70 | 6.80 | 8.20 | 9.16 | 9.80 |
|  | **2-5** | 3.70 | 4.20 | 4.92 | 6.20 | 7.50 | 8.90 | 9.50 |
|  | **6-11** | 3.30 | 3.80 | 4.60 | 5.60 | 6.90 | 8.27 | 8.80 |
|  | **12-14** | 2.40 | 2.80 | 4.00 | 5.10 | 6.40 | 7.50 | 8.30 |
|  | **15-18** | 3.60 | 3.74 | 4.40 | 6.30 | 7.70 | 8.46 | 8.88 |
| **Neutrophils** | **˂2** | 20.70 | 24.50 | 31.40 | 40.40 | 52.45 | 62.60 | 70.00 |
| **%** | **2-5** | 27.31 | 32.61 | 40.60 | 50.00 | 59.52 | 69.09 | 75.76 |
|  | **6-11** | 32.70 | 36.26 | 44.10 | 52.20 | 60.00 | 70.24 | 73.00 |
|  | **12-14** | 32.64 | 36.50 | 44.20 | 51.50 | 63.70 | 70.60 | 74.28 |
|  | **15-18** | 38.70 | 43.24 | 48.10 | 54.90 | 62.50 | 67.48 | 69.80 |
| **Lymphocytes** | **˂2** | 21.21 | 26.38 | 35.85 | 47.70 | 56.00 | 63.00 | 67.12 |
| **%** | **2-5** | 17.05 | 22,41 | 29,50 | 38,85 | 48,07 | 55,99 | 61,09 |
|  | **6-11** | 17.00 | 20.72 | 28.60 | 37.00 | 44.30 | 53.70 | 57.84 |
|  | **12-14** | 16.98 | 19.90 | 27.30 | 36.40 | 44.80 | 51.68 | 56.60 |
|  | **15-18** | 19.74 | 23.94 | 26.80 | 32.00 | 42.00 | 47.18 | 51.58 |
| **Monocytes** | **˂2** | 2.88 | 4.00 | 6.15 | 8.30 | 10.10 | 12.96 | 15.03 |
| **%** | **2-5** | 3.00 | 4.40 | 6.50 | 8.00 | 10.00 | 11.80 | 13.20 |
|  | **6-11** | 4.33 | 5.30 | 6.60 | 7.90 | 9.50 | 11.07 | 12.19 |
|  | **12-14** | 5.00 | 5.96 | 6.80 | 8.20 | 9.90 | 11.42 | 12.80 |
|  | **15-18** | 4.50 | 5.76 | 6.90 | 8.40 | 9.90 | 11.02 | 12.58 |
| **Eosinophils**  **%** | **˂2** | 0.00 | 0.00 | 0.70 | 1.50 | 2.70 | 4.00 | 5.00 |
|  | **2-5** | 0.00 | 0.10 | 0.80 | 1.30 | 2.10 | 3.20 | 4.30 |
|  | **6-11** | 0.10 | 0.40 | 0.80 | 1.30 | 2.00 | 3.00 | 3.70 |
|  | **12-14** | 0.10 | 0.40 | 0.80 | 1.20 | 2.00 | 3.36 | 4.46 |
|  | **15-18** | 0.12 | 0.20 | 0.50 | 0.90 | 1.40 | 1.86 | 2.50 |
| **Basophils**  **%** | **˂2** | 0.00 | 0.00 | 0.00 | 0.60 | 1.00 | 1.70 | 2.00 |
|  | **2-5** | 0.00 | 0.00 | 0.50 | 0.70 | 1.00 | 1.40 | 2.00 |
|  | **6-11** | 0.00 | 0.30 | 0.50 | 0.80 | 1.00 | 1.30 | 1.60 |
|  | **12-14** | 0.00 | 0.40 | 0.60 | 0.80 | 1.10 | 1.40 | 1.52 |
|  | **15-18** | 0.30 | 0.40 | 0.50 | 0.70 | 0.90 | 1.10 | 1.18 |
| **Neutrophils** 10˄3/μL | **˂2** | 1.08 | 1.35 | 1.91 | 2.71 | 3.74 | 5.11 | 6.01 |
|  | **2-5** | 1.38 | 1.64 | 2.11 | 3.03 | 4.03 | 5.37 | 6.38 |
|  | **6-11** | 1.40 | 1.66 | 2.14 | 2.77 | 3.82 | 4.99 | 5.91 |
|  | **12-14** | 1.00 | 1.16 | 1.72 | 2.62 | 3.53 | 5.00 | 5.70 |
|  | **15-18** | 1.50 | 1.72 | 2.18 | 3.30 | 4.34 | 5.52 | 6.10 |
| **Lymphocytes**  10˄3/μL | **˂2** | 1.49 | 1.76 | 2.23 | 2.98 | 3.83 | 4.66 | 5.14 |
|  | **2-5** | 1.01 | 1.27 | 1.71 | 2.27 | 2.92 | 3.70 | 4.20 |
|  | **6-11** | 0.89 | 1.07 | 1.46 | 1.96 | 2.54 | 3.25 | 4.08 |
|  | **12-14** | 0.84 | 0.97 | 1.19 | 1.69 | 2.13 | 2.94 | 3.20 |
|  | **15-18** | 1.16 | 1.36 | 1.64 | 1.96 | 2.36 | 2.85 | 3.07 |
| **Monocytes**  10˄3/μL | **˂2** | 0.17 | 0.27 | 0.40 | 0.54 | 0.73 | 0.92 | 1.06 |
|  | **2-5** | 0.20 | 0.27 | 0.36 | 0.48 | 0.62 | 0.77 | 0.92 |
|  | **6-11** | 0.22 | 0.26 | 0.33 | 0.43 | 0.56 | 0.70 | 0.77 |
|  | **12-14** | 0.16 | 0.20 | 0.30 | 0.40 | 0.50 | 0.61 | 0.75 |
|  | **15-18** | 0.23 | 0.32 | 0.38 | 0.44 | 0.66 | 0.84 | 0.95 |
| **Eosinophils**  10˄3/μL | **˂2** | 0.00 | 0.00 | 0.04 | 0.09 | 0.18 | 0.27 | 0.34 |
|  | **2-5** | 0.00 | 0.01 | 0.04 | 0.08 | 0.13 | 0.20 | 0.28 |
|  | **6-11** | 0.01 | 0.02 | 0.04 | 0.07 | 0.12 | 0.18 | 0.23 |
|  | **12-14** | 0.01 | 0.02 | 0.03 | 0.05 | 0.11 | 0.19 | 0.26 |
|  | **15-18** | 0.01 | 0.01 | 0.02 | 0.05 | 0.08 | 0.14 | 0.18 |
| **Basophils**  10˄3/μL | **˂2** | 0.00 | 0.00 | 0.00 | 0.04 | 0.07 | 0.11 | 0.17 |
|  | **2-5** | 0.00 | 0.00 | 0.03 | 0.04 | 0.06 | 0.09 | 0.13 |
|  | **6-11** | 0.00 | 0.01 | 0.03 | 0.04 | 0.06 | 0.08 | 0.09 |
|  | **12-14** | 0.00 | 0.02 | 0.03 | 0.03 | 0.06 | 0.08 | 0.09 |
|  | **15-18** | 0.02 | 0.02 | 0.03 | 0.03 | 0.05 | 0.07 | 0.08 |
| **Platelets**  10˄3/μL | **˂2** | 196.0 | 212.80 | 267.0 | 323.0 | 382.50 | 435.60 | 468.9 |
|  | **2-5** | 194.0 | 220.10 | 258.0 | 305.0 | 354.0 | 404.0 | 429.0 |
|  | **6-11** | 200.00 | 211.30 | 247.0 | 287.0 | 337.0 | 384.70 | 414.85 |
|  | **12-14** | 185.20 | 199.40 | 229.0 | 271.0 | 312.0 | 350.60 | 375.40 |
|  | **15-18** | 177.40 | 184.80 | 226.0 | 264.0 | 306.0 | 331.60 | 396.40 |
| **MPV**  **fL** | **˂2** | 6.50 | 6.80 | 7.20 | 7.70 | 8.30 | 8.90 | 9.20 |
|  | **2-5** | 6.40 | 6.60 | 6.90 | 7.40 | 7.90 | 8.40 | 8.70 |
|  | **6-11** | 6.40 | 6.70 | 7.10 | 7.60 | 7.90 | 8.30 | 8.60 |
|  | **12-14** | 6.68 | 6.70 | 7.20 | 7.70 | 8.10 | 8.44 | 8.70 |
|  | **15-18** | 6.72 | 6.80 | 7.30 | 7.70 | 8.20 | 8.56 | 9.72 |

RDW = red cell distribution width, MCV = mean corpuscular volume, MCH = mean corpuscular hemoglobin, MCHC = mean corpuscular hemoglobin concentration, MPV = mean platelet volume.

**Table S2. Female Percentiles**

| **Indicator** | **Age (years)** | **Percentile** | | | | | | |
| --- | --- | --- | --- | --- | --- | --- | --- | --- |
| **Hemoglobin** |  | **5** | **10** | **25** | **50** | **75** | **90** | **95** |
| g/dL | **˂2** | 10.36 | 10.82 | 12.00 | 13.20 | 14.20 | 15.00 | 15.44 |
|  | **2-5** | 12.30 | 12.60 | 13.30 | 14.10 | 14.90 | 15.50 | 15.80 |
|  | **6-11** | 13.20 | 13.53 | 14.10 | 14.80 | 15.40 | 16.10 | 16.40 |
|  | **12-14** | 13.38 | 14.00 | 14.70 | 15.20 | 15.70 | 16.30 | 16.71 |
|  | **15-18** | 13.66 | 14.10 | 14.65 | 15.40 | 15.90 | 16.40 | 16.52 |
| **Hematocrit** | **˂2** | 30.04 | 32.80 | 36.10 | 39.10 | 42.10 | 45.28 | 46.9 |
| **%** | **2-5** | 36.20 | 37.50 | 39.30 | 41.60 | 43.70 | 45.70 | 47.00 |
|  | **6-11** | 38.61 | 40.00 | 41.60 | 43.40 | 45.40 | 47.17 | 48.00 |
|  | **12-14** | 39.27 | 41.01 | 43.12 | 44.90 | 46.67 | 48.16 | 49.21 |
|  | **15-18** | 41.16 | 42.08 | 43.25 | 45.30 | 47.10 | 48.40 | 49.13 |
| ***Erythrocytes*** | **˂2** | 3.21 | 3.57 | 3.92 | 4.30 | 4.71 | 5.09 | 5.35 |
| 10^˄^6/μL | **2-5** | 3.87 | 4.06 | 4.28 | 4.52 | 4.82 | 5.02 | 5.16 |
|  | **6-11** | 4.01 | 4.21 | 4.42 | 4.63 | 4.87 | 5.03 | 5.16 |
|  | **12-14** | 4.14 | 4.26 | 4.47 | 4.68 | 4,91 | 5.11 | 5.21 |
|  | **15-18** | 4.18 | 4.39 | 4.51 | 4.68 | 4.89 | 5.09 | 5.14 |
| **RDW** | **˂2** | 13.16 | 13.40 | 14.20 | 15.20 | 17.20 | 19.90 | 22.14 |
| **%** | **2-5** | 13.10 | 13.20 | 13.70 | 14.30 | 15.20 | 16.40 | 17.5 |
|  | **6-11** | 12.90 | 13.10 | 13.40 | 13.90 | 14.40 | 15.27 | 15.80 |
|  | **12-14** | 13.00 | 13.10 | 13.60 | 13.90 | 14.40 | 14.93 | 15.53 |
|  | **15-18** | 12.99 | 13.10 | 13.40 | 13.80 | 14.30 | 14.80 | 15.27 |
| **MCV** | **˂2** | 81.00 | 84.32 | 87.90 | 91.00 | 94.60 | 99.30 | 100.88 |
| **fL** | **2-5** | 85.10 | 86.60 | 88.70 | 91.70 | 94.70 | 97.70 | 99.20 |
|  | **6-11** | 88.20 | 89.13 | 91.50 | 94.30 | 96.20 | 98.00 | 99.00 |
|  | **12-14** | 88.91 | 91.01 | 93.15 | 95.40 | 97.87 | 100.73 | 101.31 |
|  | **15-18** | 90.57 | 91.74 | 94.10 | 96.90 | 98.45 | 100.72 | 101.80 |
| **MCH**  **pg** | **˂2** | 26.38 | 27.72 | 29.30 | 30.40 | 31.90 | 33.10 | 33.84 |
|  | **2-5** | 28.30 | 29.00 | 30.00 | 31.00 | 32.20 | 33.40 | 33.90 |
|  | **6-11** | 29.30 | 30.00 | 31.00 | 32.00 | 32.90 | 33.70 | 34.30 |
|  | **12-14** | 29.78 | 30.57 | 31.52 | 32.30 | 33.47 | 34.70 | 35.24 |
|  | **15-18** | 30.26 | 30.78 | 31.70 | 32.50 | 33.60 | 34.40 | 34.71 |
| **MCHC**  **g/dL** | **˂2** | 31.36 | 31.90 | 32.8 | 33.40 | 34.00 | 34.60 | 35.10 |
|  | **2-5** | 32.30 | 32.80 | 33.20 | 33.80 | 34.40 | 35.00 | 35.30 |
|  | **6-11** | 32.90 | 33.13 | 33.60 | 34.00 | 34.50 | 34.94 | 35.40 |
|  | **12-14** | 32.80 | 33.10 | 33.50 | 33.90 | 34.37 | 34.90 | 35.43 |
|  | **15-18** | 32.67 | 33.20 | 33.40 | 33.80 | 34.20 | 34.60 | 34.80 |
| **Leukocytes** 10˄3/μL | **˂2** | 3.90 | 4.40 | 5.40 | 6.80 | 8.10 | 9.30 | 9.80 |
|  | **2-5** | 3.60 | 4.00 | 4.70 | 5.80 | 7.20 | 8.40 | 9.20 |
|  | **6-11** | 3.41 | 3.70 | 4.20 | 5.10 | 6.49 | 7.80 | 8.79 |
|  | **12-14** | 3.77 | 4.10 | 4.70 | 5.70 | 7.37 | 8.86 | 9.73 |
|  | **15-18** | 3.88 | 4.28 | 5.10 | 6.00 | 7.60 | 8.42 | 9.41 |
| **Neutrophils** | **˂2** | 17.88 | 23.24 | 29.90 | 40.30 | 52.50 | 65.48 | 74.00 |
| **%** | **2-5** | 28.20 | 32.30 | 40.00 | 48.10 | 59.00 | 70.00 | 76.00 |
|  | **6-11** | 35.83 | 39.13 | 46.17 | 53.40 | 60.92 | 70.49 | 76.10 |
|  | **12-14** | 37.93 | 42.37 | 50.72 | 56.20 | 65.00 | 75.83 | 80.01 |
|  | **15-18** | 37.78 | 41.26 | 51.15 | 59.20 | 67.25 | 71.08 | 74.03 |
| **Lymphocytes** | **˂2** | 19.60 | 25.06 | 36.80 | 47.90 | 58.00 | 65.00 | 69.76 |
| **%** | **2-5** | 16.50 | 22.00 | 31.00 | 41.00 | 49.00 | 57.00 | 61.50 |
|  | **6-11** | 17.03 | 20.20 | 27.87 | 35.90 | 43.62 | 50.51 | 53.65 |
|  | **12-14** | 12.61 | 15.34 | 24.40 | 33.00 | 39.12 | 47.64 | 53.22 |
|  | **15-18** | 16.34 | 19.42 | 23.45 | 31.20 | 40.90 | 48.24 | 51.31 |
| **Monocytes** | **˂2** | 2.00 | 4.00 | 6,00 | 8.30 | 10.30 | 13.82 | 16.20 |
| **%** | **2-5** | 3.00 | 4.60 | 5.90 | 7.50 | 9.50 | 11.20 | 12.70 |
|  | **6-11** | 4.01 | 5.20 | 6.30 | 7.50 | 8.92 | 10.70 | 11.98 |
|  | **12-14** | 4.37 | 5.90 | 6.42 | 7.65 | 8.90 | 10.00 | 11.00 |
|  | **15-18** | 4.37 | 5.78 | 6..50 | 7.60 | 8.55 | 9.20 | 10.73 |
| **Eosinophils**  **%** | **˂2** | 0.00 | 0.00 | 0.20 | 1.10 | 2.00 | 3.10 | 4.32 |
|  | **2-5** | 0.00 | 0.00 | 0.60 | 1.20 | 2.00 | 3.00 | 4.00 |
|  | **6-11** | 0.00 | 0.40 | 0.70 | 1.00 | 1.90 | 3.07 | 3.88 |
|  | **12-14** | 0.28 | 0.50 | 0.80 | 1.30 | 1.80 | 2.73 | 3.34 |
|  | **15-18** | 0.18 | 0.38 | 0.70 | 1.30 | 1.95 | 2.82 | 4.25 |
| **Basophils**  **%** | **˂2** | 0.00 | 0.00 | 0.00 | 0.60 | 1.0 | 1.60 | 2.14 |
|  | **2-5** | 0.00 | 0.00 | 0.40 | 0.60 | 0.90 | 1.30 | 1.90 |
|  | **6-11** | 0.00 | 0.30 | 0.50 | 0.70 | 1.0 | 1.20 | 1.40 |
|  | **12-14** | 0.00 | 0.30 | 0.40 | 0.70 | 0.90 | 1.20 | 1.51 |
|  | **15-18** | 0.00 | 0.38 | 0.50 | 0.70 | 0.90 | 1.14 | 1.51 |
| **Neutrophils** 10˄3/μL | **˂2** | 0.89 | 1.29 | 1.75 | 2.65 | 3.94 | 5.23 | 5.86 |
|  | **2-5** | 1.34 | 1.55 | 1.94 | 2.65 | 3.84 | 5.55 | 6.26 |
|  | **6-11** | 1.43 | 1.62 | 1.99 | 2.68 | 3.70 | 5.10 | 6.04 |
|  | **12-14** | 1.71 | 2.05 | 2.48 | 3.12 | 4.28 | 6.02 | 6.45 |
|  | **15-18** | 1.67 | 1.96 | 2.54 | 3.59 | 4.71 | 5.79 | 6.10 |
| **Lymphocytes**  10˄3/μL | **˂2** | 1.08 | 1.55 | 2.39 | 3.06 | 3.89 | 4.67 | 5.40 |
|  | **2-5** | 1.12 | 1.37 | 1.68 | 2.16 | 2.85 | 3.65 | 4.00 |
|  | **6-11** | 0.88 | 1.10 | 1.37 | 1.79 | 2.29 | 2.87 | 3.18 |
|  | **12-14** | 0.89 | 0.99 | 1.30 | 1.70 | 2.33 | 3.48 | 3.75 |
|  | **15-18** | 0.98 | 1.16 | 1.41 | 1.98 | 2.31 | 2.64 | 3.32 |
| **Monocytes**  10˄3/μL | **˂2** | 0.13 | 0.22 | 0.38 | 0.55 | 0.74 | 0.94 | 1.15 |
|  | **2-5** | 0.18 | 0.23 | 0.32 | 0.43 | 0.55 | 0.70 | 0.78 |
|  | **6-11** | 0.22 | 0.25 | 0.30 | 0.40 | 0.47 | 0.59 | 0.70 |
|  | **12-14** | 0.26 | 0.30 | 0.36 | 0.42 | 0.54 | 0.64 | 0.80 |
|  | **15-18** | 0.25 | 0.31 | 0.36 | 0.45 | 0.55 | 0.64 | 0.70 |
| **Eosinophils**  10˄3/μL | **˂2** | 0.00 | 0.00 | 0.01 | 0.08 | 0.14 | 0.21 | 0.31 |
|  | **2-5** | 0.00 | 0.00 | 0.03 | 0.07 | 0.11 | 0.17 | 0.24 |
|  | **6-11** | 0.00 | 0.02 | 0.03 | 0.06 | 0.10 | 0.16 | 0.19 |
|  | **12-14** | 0.02 | 0.03 | 0.05 | 0.07 | 0.10 | 0.14 | 0.19 |
|  | **15-18** | 0.01 | 0.02 | 0.04 | 0.08 | 0.12 | 0.18 | 0.21 |
| **Basophils**  10˄3/μL | **˂2** | 0.00 | 0.00 | 0.00 | 0.04 | 0.07 | 0.10 | 0.17 |
|  | **2-5** | 0.00 | 0.00 | 0.02 | 0.03 | 0.05 | 0.08 | 0.10 |
|  | **6-11** | 0.00 | 0.01 | 0.02 | 0.03 | 0.05 | 0.07 | 0.08 |
|  | **12-14** | 0.00 | 0.02 | 0.03 | 0.04 | 0.05 | 0.07 | 0.08 |
|  | **15-18** | 0.0 | 0.02 | 0.03 | 0.04 | 0.05 | 0.07 | 0.10 |
| **Platelets**  10˄3/μL | **˂2** | 158.60 | 184.40 | 249.00 | 318.00 | 374.00 | 415.60 | 456.80 |
|  | **2-5** | 177.00 | 204.00 | 247.00 | 297.00 | 349.00 | 405.00 | 432.00 |
|  | **6-11** | 182,15 | 197.00 | 229.75 | 275.50 | 320.00 | 364.00 | 397.00 |
|  | **12-14** | 169.00 | 186.50 | 215.00 | 245.50 | 301.75 | 346.20 | 393.75 |
|  | **15-18** | 169.40 | 173.80 | 204.00 | 245.00 | 305.50 | 347.80 | 381.40 |
| **MPV**  **fL** | **˂2** | 6.50 | 6.70 | 7.10 | 7.70 | 8.40 | 9.00 | 9.50 |
|  | **2-5** | 6.30 | 6.50 | 6.90 | 7.40 | 7.80 | 8.30 | 8.60 |
|  | **6-11** | 6.40 | 6.60 | 7.00 | 7.30 | 7.80 | 8.20 | 8.50 |
|  | **12-14** | 6.60 | 6.90 | 7.20 | 7.50 | 7.90 | 8.33 | 8.60 |
|  | **15-18** | 6.79 | 6.90 | 7.15 | 7.60 | 8.00 | 8.50 | 8.71 |

RDW = red cell distribution width, MCV = mean corpuscular volume, MCH = mean corpuscular hemoglobin, MCHC = mean corpuscular hemoglobin concentration, MPV = mean platelet volume.
