# Supplementary figures and images for: Blood cytology in children with down syndrome
Source: BMC Pediatr. 2022 Jul 2;22:387. doi: 10.1186/s12887-022-03450-8 (PMC9250261; doi:10.1186/s12887-022-03450-8)

FIGURE S1. HEMATOLOGICAL PARAMETERS BY AGE AND SEX


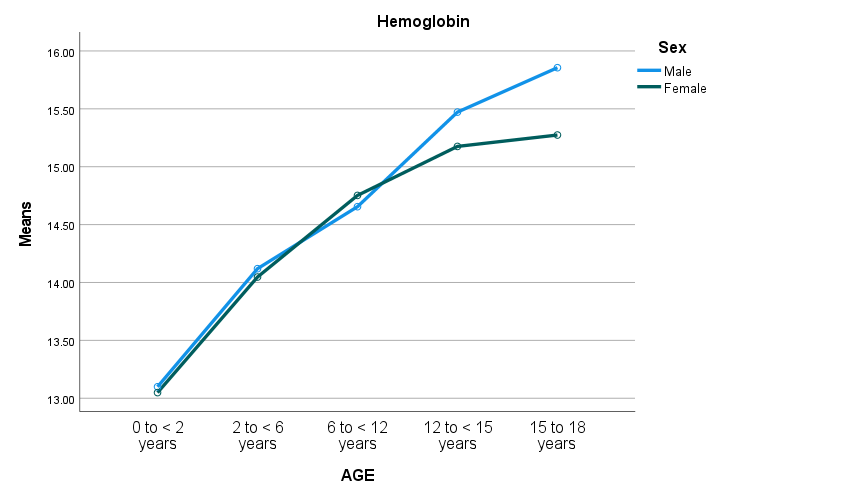


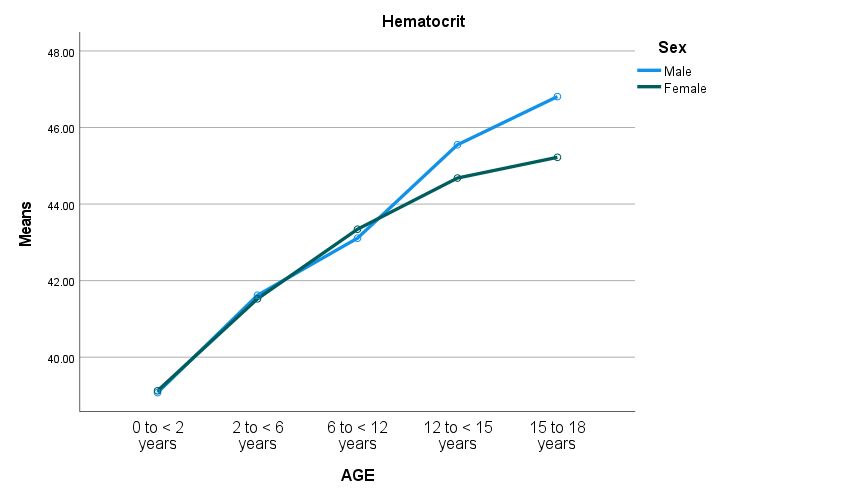


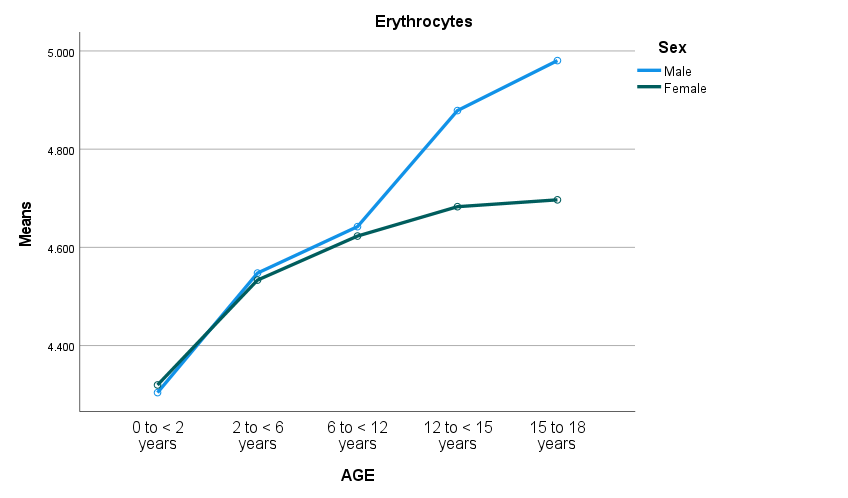


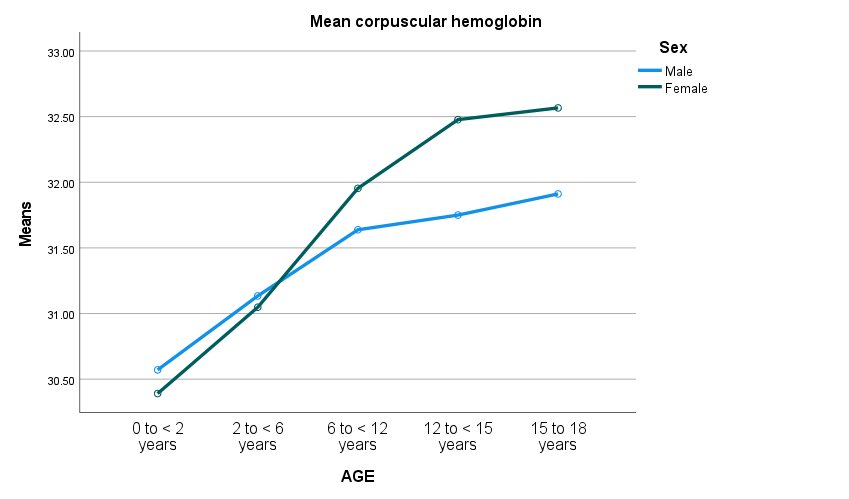


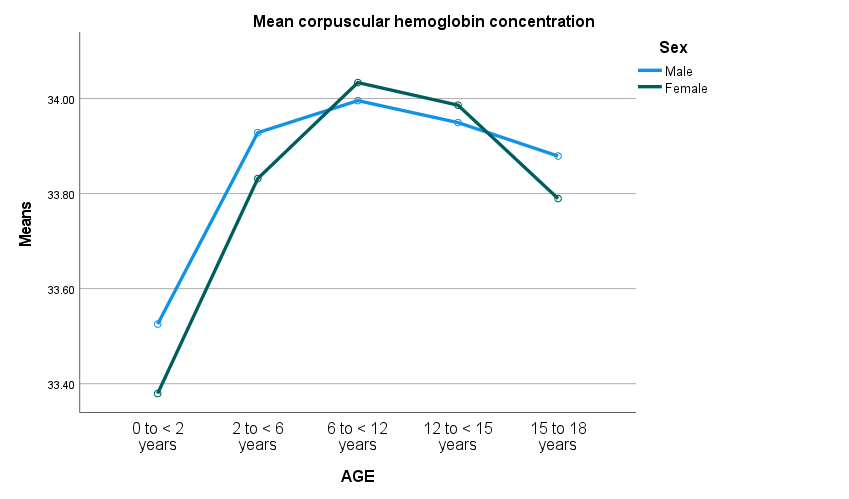


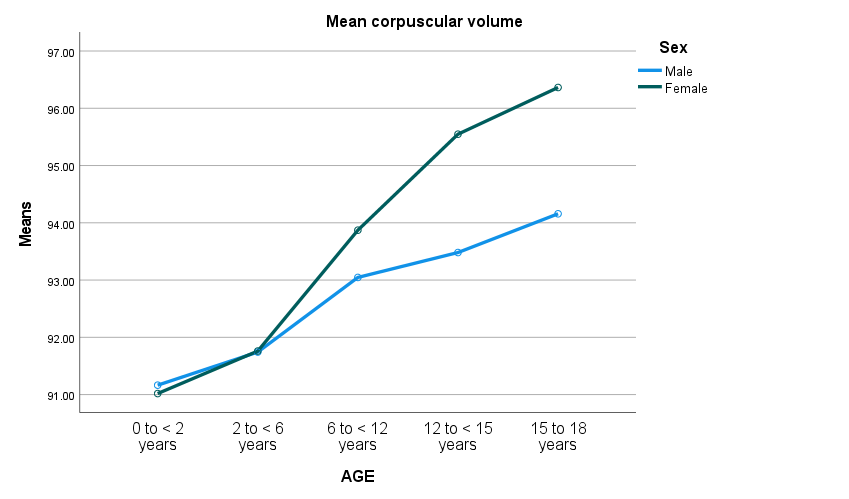


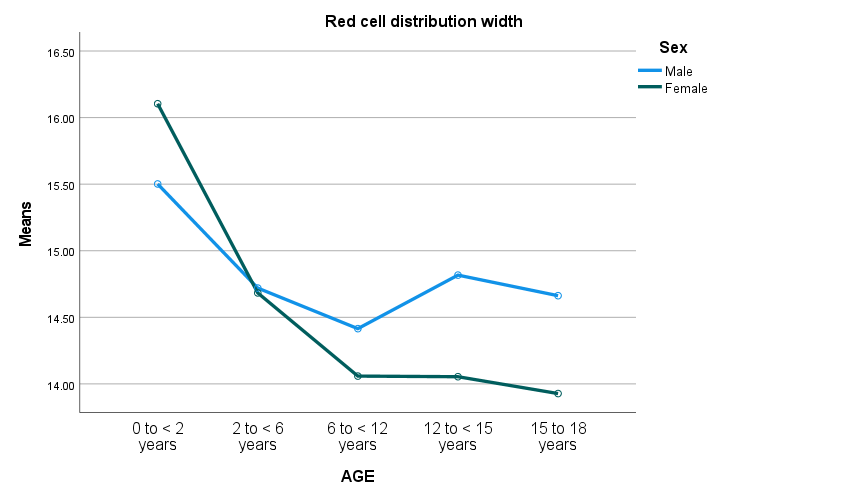


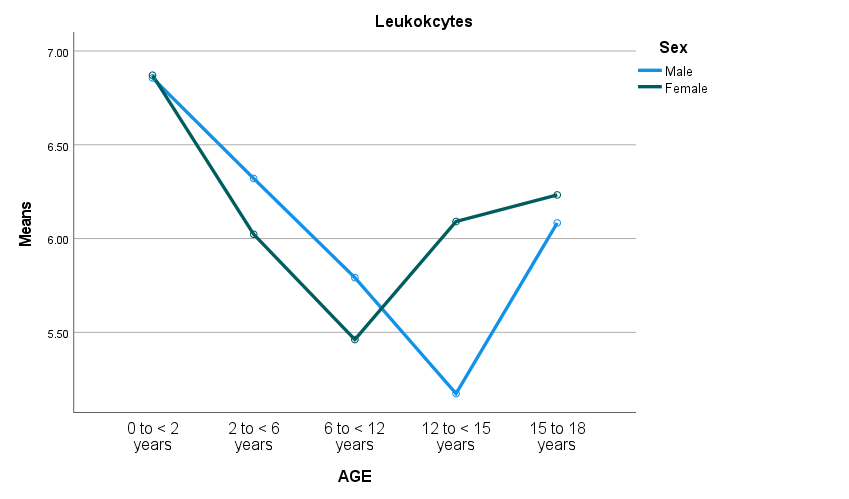


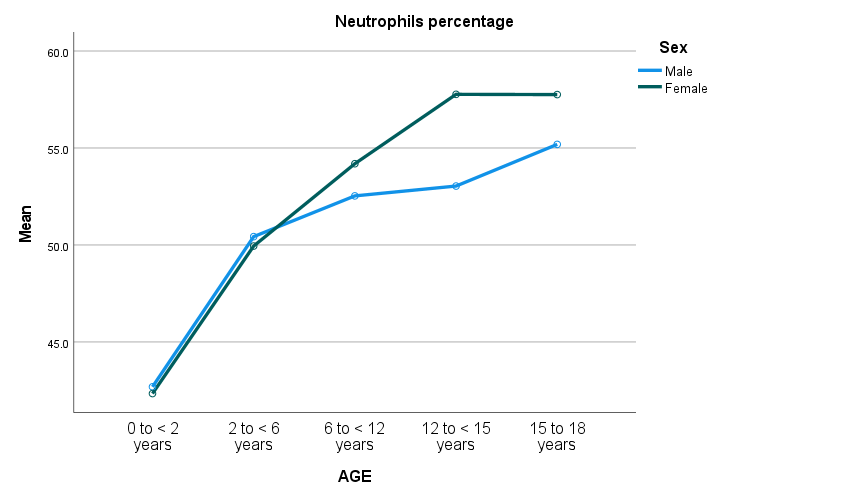


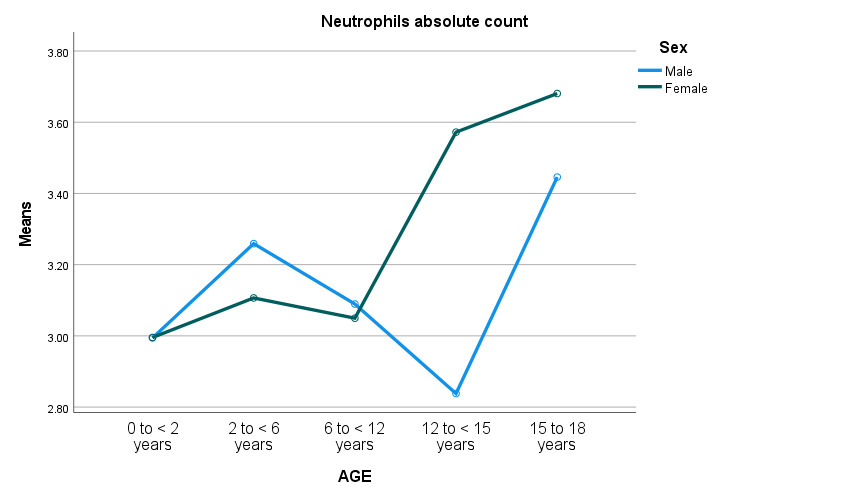


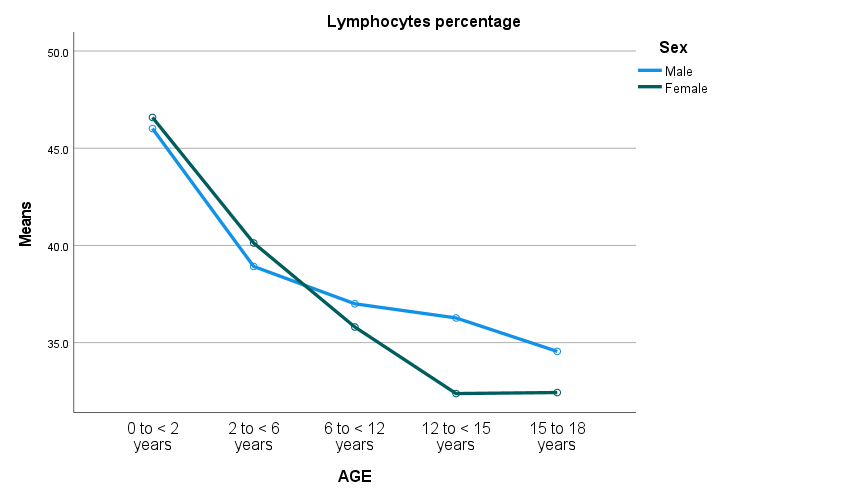


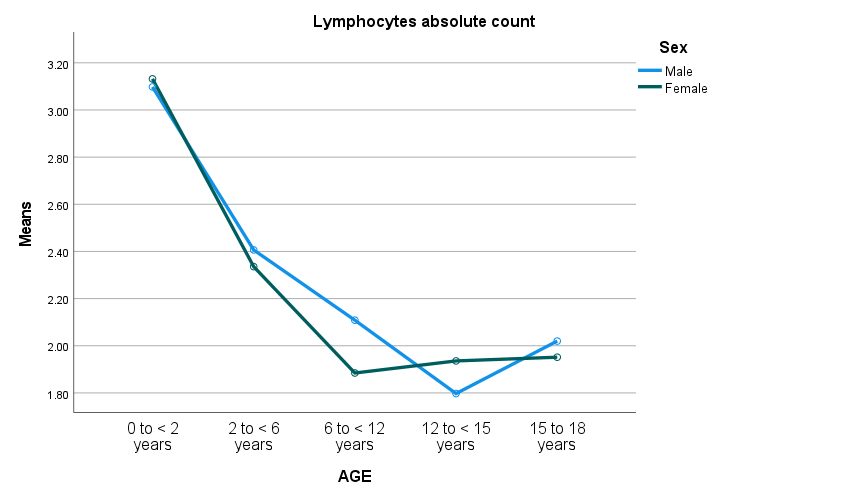


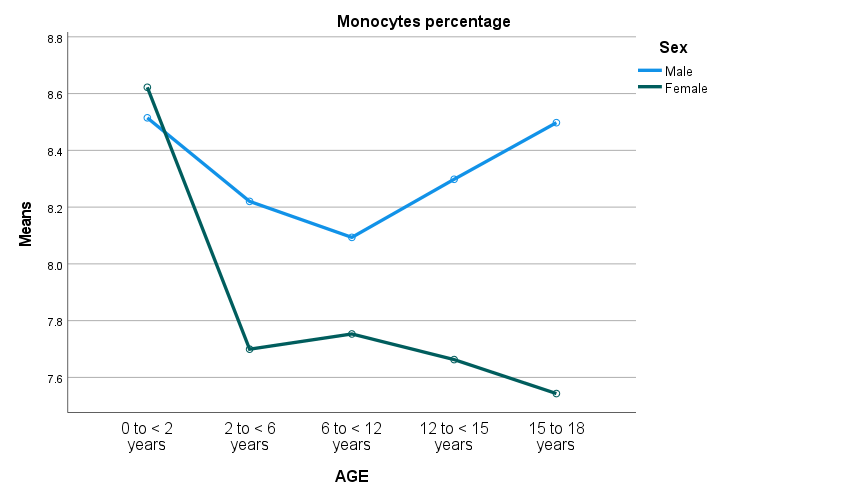


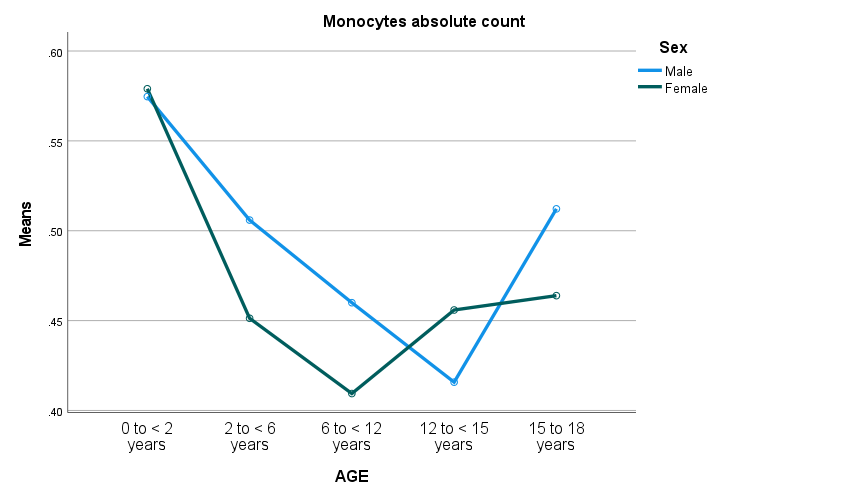


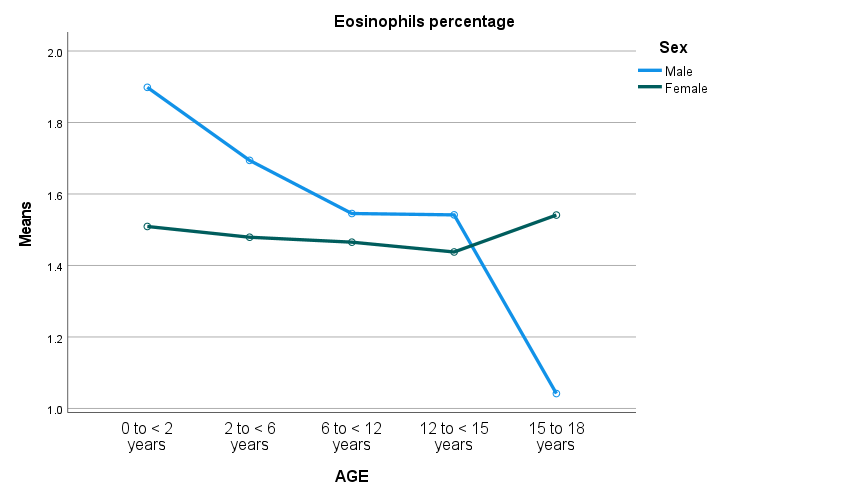


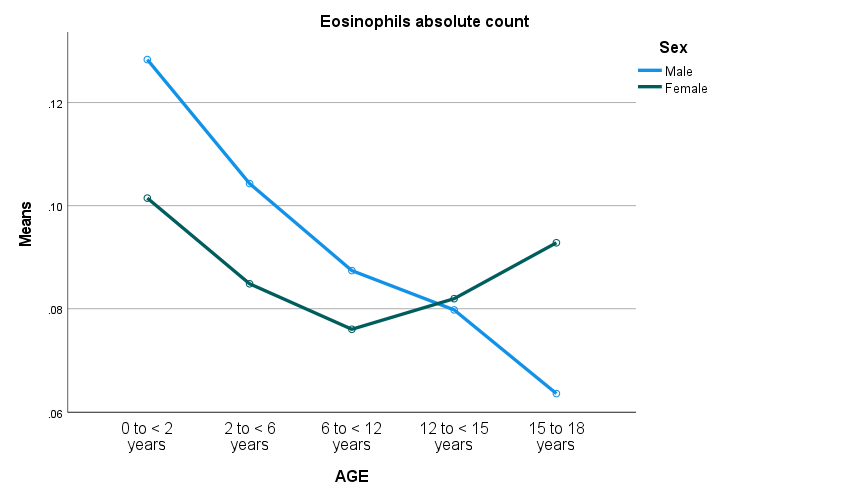


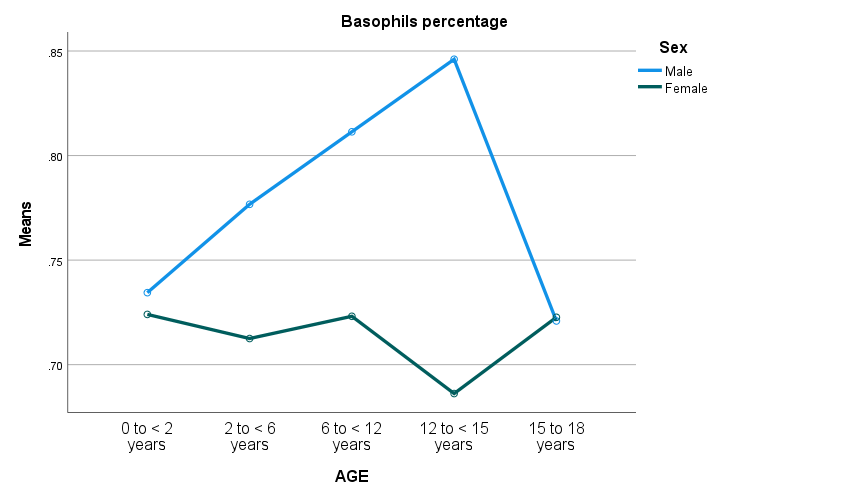


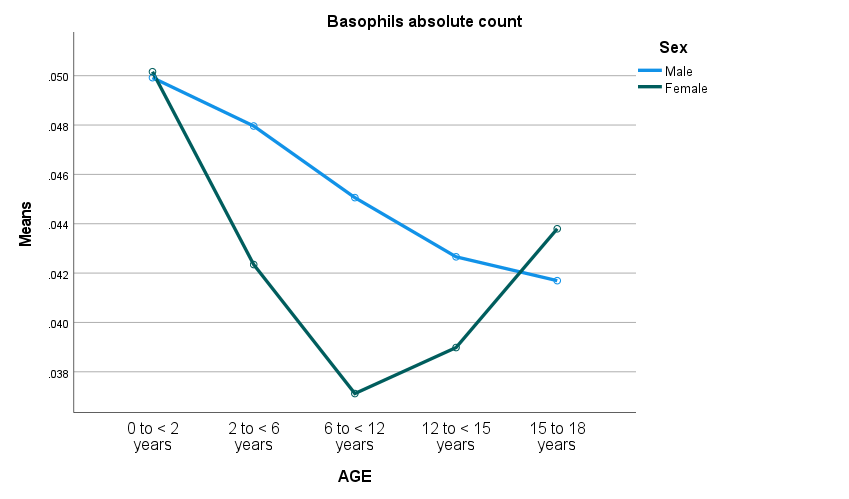


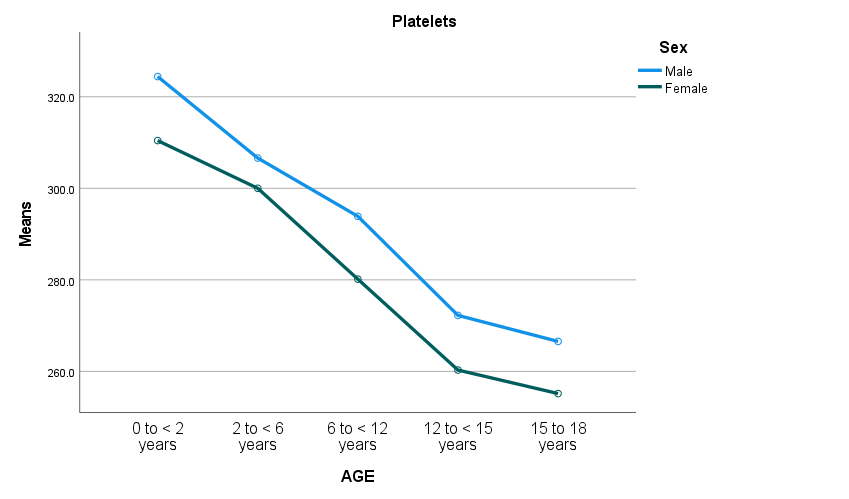


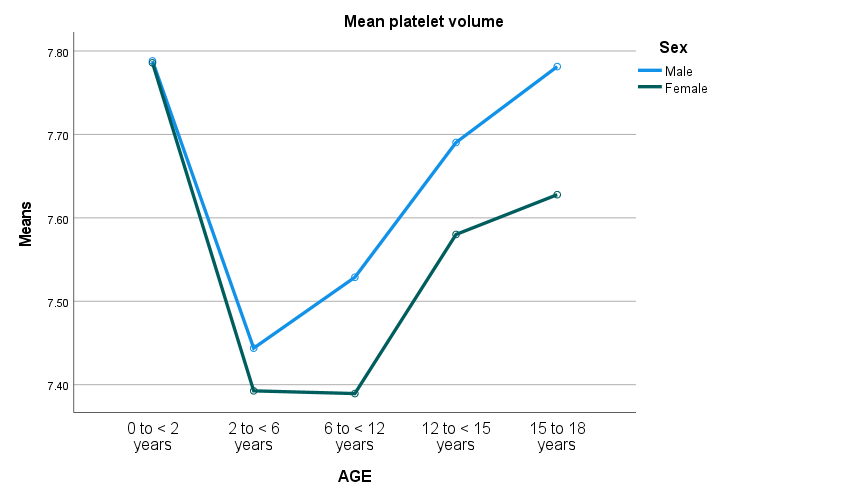

Supplement: Supplementary file 2 — Additional file 2. [file 12887_2022_3450_MOESM2_ESM.docx]
